# Clinical characteristics, treatment and prognosis of infants with retinoblastoma: a multicenter, 10-year retrospective analysis

**DOI:** 10.1186/s12887-023-03984-5

**Published:** 2023-05-09

**Authors:** Yi Zhang, Yizhuo Wang, Tian Zhi, Mei Jin, Dongsheng Huang, Xiaoli Ma

**Affiliations:** 1 Department of Pediatrics, Beijing Tongren Hospital, Capital Medical University, Beijing, 100730 China; 2 Department of Medical Oncology, Beijing Children’s Hospital, Capital Medical University, National Center for Children’s Health, Beijing, 100045 China

**Keywords:** Retinoblastoma, Comprehensive treatment, Overall survival rate, Recurrence, Prognosis

## Abstract

**Background:**

To summarize the characteristics and treatment, and analyze the prognosis of large number of infants with retinoblastoma (RB) in China through a multicenter, 10-year retrospective analysis.

**Methods:**

The data of RB infants were collected from multiple centers. The characteristics and survival prognosis were analyzed. The overall survival (OS) rate was estimated by the Kaplan–Meier method. Multivariate Cox survival analysis was to evaluate the independent risk factors affecting the prognosis of RB infants.

**Results:**

A total of 373 RB infants (202 boys and 171 girls) were included, the median age was 6.22 months (10 days to 11.93 months). The median follow-up time of RB infants was 18.4 (1.02–122.81 months). After excluding the lost to follow-up cases, the OS rate was 97.7% (345/353). Kaplan–Meier survival analysis indicated that 9 cases died and the median survival time was not reached. Univariate analysis of prognostic factors revealed eye affected, presenting signs, left eye stage and recurrence to be poor prognostic factors for OS rate in RB infants (all *P* < 0.05). Multivariate Cox regression analyses for OS showed recurrence (HR = 1.376, 95% CI: 0.878–2.156, *P* = 0.048) was an independent factor for prognosis of infants with RB. The median survival time of infants underwent chemotherapy + intra-arterial chemotherapy (IAC) + enucleation + vitrectomy was the longest than other treatments (*n* = 9, 47.64 months, OS = 100%, all *P* < 0.05). There was a history of RB in 17 infants' lineal relatives. Kaplan-merier survival analysis indicated 1 case died and the median survival time was not reached.

**Conclusion:**

Recurrence is an independent factor for prognosis of RB infants, which still needs attention after treatment. Early screening, comprehensive treatments and follow-up of patients may lead to improvements of prognosis of RB infants.

## Background

Retinoblastoma (RB) is the most common malignant tumor in children, and its incidence ranks the third in all age groups of ocular malignancies [1]. The incidence rate of RB was at 1 in 15,000–20,000 live birth across population, corresponding to about 9000 new cases every year [2]. China is the most populous country in the world, with about 1000 new cases of RB every year, accounting for 1/8 of all cases worldwide [3, 4]. It originates from primitive retinal stem cells or cone precursors, 95% of cases occur before 5 years old, the median age of onset is 2–3 years old [5]. Infants with infantile disease may become seriously ill shortly after birth, either in one eye or in both eyes, with either endophytic or exophytic growth [6]. Leukocoria is the most common initial presenting sign of RB which is seen in 60 to 80% of newly diagnosed patients, followed by strabismus [7].

The younger the onset age of RB, the higher the incidence rate of RB in both eyes, and it is not easy to detect and diagnose early [8]. It is clear that RB has a significant genetic tendency, the clear family history of RB is about for 5% of the children with the disease in China [9]. With the improvement of early diagnosis, the treatment of RB patients has been improved. The treatment of RB depends on the age of the child, laterality, and degree of disease. There are several treatment modalities for RB, such as chemotherapy, brachytherapy, external beam radiotherapy, enucleation, and intra-arterial chemotherapy (IAC) [2]. Moreover, the treatment of infant RB is different from that of other age groups. For example, there is no clear conclusion on whether the timing of enucleation of eyeball, whether the prosthesis is installed at the same time or delayed plastic installation after enucleation of eyeball, whether it can tolerate systemic chemotherapy, and whether intraocular chemotherapy is applicable to infants within 6 months.

Patients with advanced RB usually have poor prognosis. Prognosis and survival depends on early diagnosis and appropriate treatment. Early diagnosis, care and treatment of retinoblastoma is a challengeable issue for China health system. Therefore, in order to early detection, diagnosis, treatment, improve the cure rate and preserve the eye rate, we collected the clinical data of multi-center infants with large number of RB in china and retrospectively analyzed the characteristics, family genetic tendency, treatment and prognosis of RB patients, so as to provide theoretical basis for early screening, diagnosis and treatment of RB in the future.

## Materials and methods

### Patients

The data of 943 infants with RB from January 2010 to December 2019 were collected from multiple centers (Beijing Tongren Hospital, Capital Medical University; Beijing Children's Hospital, Capital Medical University; Peking University First Hospital; Beijing Institute of Pediatrics, Capital Medical University; Beijing Shijitan Hospital, Capital Medical University; Beijing Tiantan Hospital, Capital Medical University; Beijing Jishuitan Hospital, Capital Medical University). The inclusion criteria were as follows: 1) infants aged ≤ 12 months; 2) infants with RB were diagnosed through fundus examination using RetCam under general anesthesia, combined with B ultrasound, orbital CT/MRI for diagnosis, and/or surgical removal of the eyeball [10]; 3) All infants were treated according to the treatment plan; The exclusion criteria were as follows: 1) Combined with congenital immunodeficiency disease or secondary RB; 2) No effective follow-up information was obtained at 3 months; (3) The patients were not followed up for more than 3 times, and the diagnosis and treatment information was incomplete during shedding. This study was approved by the ethics committee of Beijing Tongren Hospital, Capital Medical University (No. 2020–06-BJRRB-ZY). Written informed consent was obtained from their guardians.

### Diagnosis and classification

The diagnosis of RB was clinically and histopathologically made by ophthalmologists and pathologists. The stage of extraocular disease was classified according to the International Retinoblastoma Staging System (IRSS) proposed by Chantada et al. [11]. Intraocular disease was classified to group A-E by the International Intraocular Classification of Retinoblastoma (IIRC) [12, 13]. Staging to determine the treatment choice in each patient was independently assessed by two expert ophthalmologists in different institutions.

### Therapy

Patients received systematic or IAC, eye enucleation and topical therapy. Multi-drug combination chemotherapy was adopted in systematic therapy on the basis of the traditional CEV regimen, including carboplatin (< 10 kg, 18.6 mg/kg; ≥ 10 kg, 560 mg/m^2^; intravenous drip on day 1), etoposide (< 10 kg, 5 mg/kg; ≥ 10 kg, 150 mg/m^2^, intravenous drip on the day 1–2), vincristine (< 10 kg, 0.05 mg/kg; ≥ 10 kg 1.5 mg/m^2^; maximum dose 2 mg; intravenous injection or drip on day 1), [14]. IAC was mainly used for infants who aged > 4 months with intraocular tumors (subretinal tumors) [15]. Drug usage and dosage: 1) Melphalan, aged 4–6 months, 2.5 mg each cycle, local injection; aged 6–12 months, 3.0 mg each cycle, local injection; diluted with 50 mL normal saline and the pumping time was 30–45 min. 2) Carboplatin, aged 4–6 months, 20 mg each cycle; aged 6–12 months: 30 mg each cycle; saline dilution, slowly pump. The chemotherapy was sustained for 4–6 cycles, and 4 weeks were one cycle of chemotherapy. Patients with extraocular RB were generally treated with intrathecal injection combined with chemotherapy, with 9–12 cycles of chemotherapy. In detail, intrathecal therapy was given on the first day of chemotherapy: methotrexate 5 mg, cytarabine 12 mg, and dexamethasone 2 mg. Toxicities and side effects of chemotherapy were evaluated according to WHO and previously published literature [16].

Intraocular topical therapy mainly included extrascleral freezing, retinal laser photocoagulation and transpupillary thermotherapy. As an adjunct to systemic comprehensive therapy, it was suitable for small tumors (less than 3–6 mm), mostly for bilateral disease patients. Vitrectomy is suitable for the patients who were not effective in other eye conservation treatments and have indications for eye conservation treatment, especially for the patients with one affected eye. Intraoperative infusion of melphalan was feasible to maintain the effective drug concentration in vitreous cavity, and postoperative systemic intravenous chemotherapy is given to reduce the risk of local spread or systemic metastasis. Indication of eye enucleation: 1) Tumor is large and full of the whole vitreous. 2) Tumor invades the anterior chamber or neovascular glaucoma occurs, and the possibility of vision preservation is very small. 3) Tumor is suspected to spread to the optic nerve, but the scope is still in the affected eye at the proximal end of the retrobulbar optic nerve. 4) Unilateral non visual retinoblastoma. 5) Some advanced cases.

### Observation index and follow-up

The characteristics of age, sex, clinical stages, onset site, family history and survival prognosis were analyzed. The median follow-up time was 18.44 (1.02–122.81 months). The prognostic index was the overall survival (OS) rate, indicating the termination events of follow-up: recurrence, death and survival. Up to March 31, 2020, 373 cases were followed up with complete clinical data. Recurrence referred to the appearance of new lesions inside and outside the eyeball after more than 3 months of treatment: intraocular recurrent tumors can not only involve the retina or uvea, but also can be independent disseminated lesions; new lesions in distant tissues and organs outside the eyeball were identified as metastatic tumors by imaging/histopathology.

### Statistical analysis

All analysis was performed using SPSS 20.0 software. The counting data were expressed in rate (%). Chi-square and Fisher test were used to evaluate the difference between groups. Independent sample t-test and rank sum test were used to compare continuous variables. Univariate variables included gender, initial symptoms, age group (< 6 months and 7–12 months), family history, affected eye (unilateral, bilateral), intraocular stage, and recurrence. Univariate variables were analyzed mainly with prognostic outcomes, and analyzed by Kaplan-Merier survival analysis and Log-rank test to predictor the risk factors affecting prognosis of infants with RB. With the variables that were significant in the univariate analysis (*P* < 0.10), we then performed Multivariate Cox survival analysis to evaluate the independent risk factors affecting the prognosis of infants with RB. A value of *P* < 0.05 was considered statistically significant.

## Results

### Baseline characteristics

**Fig. 1:**
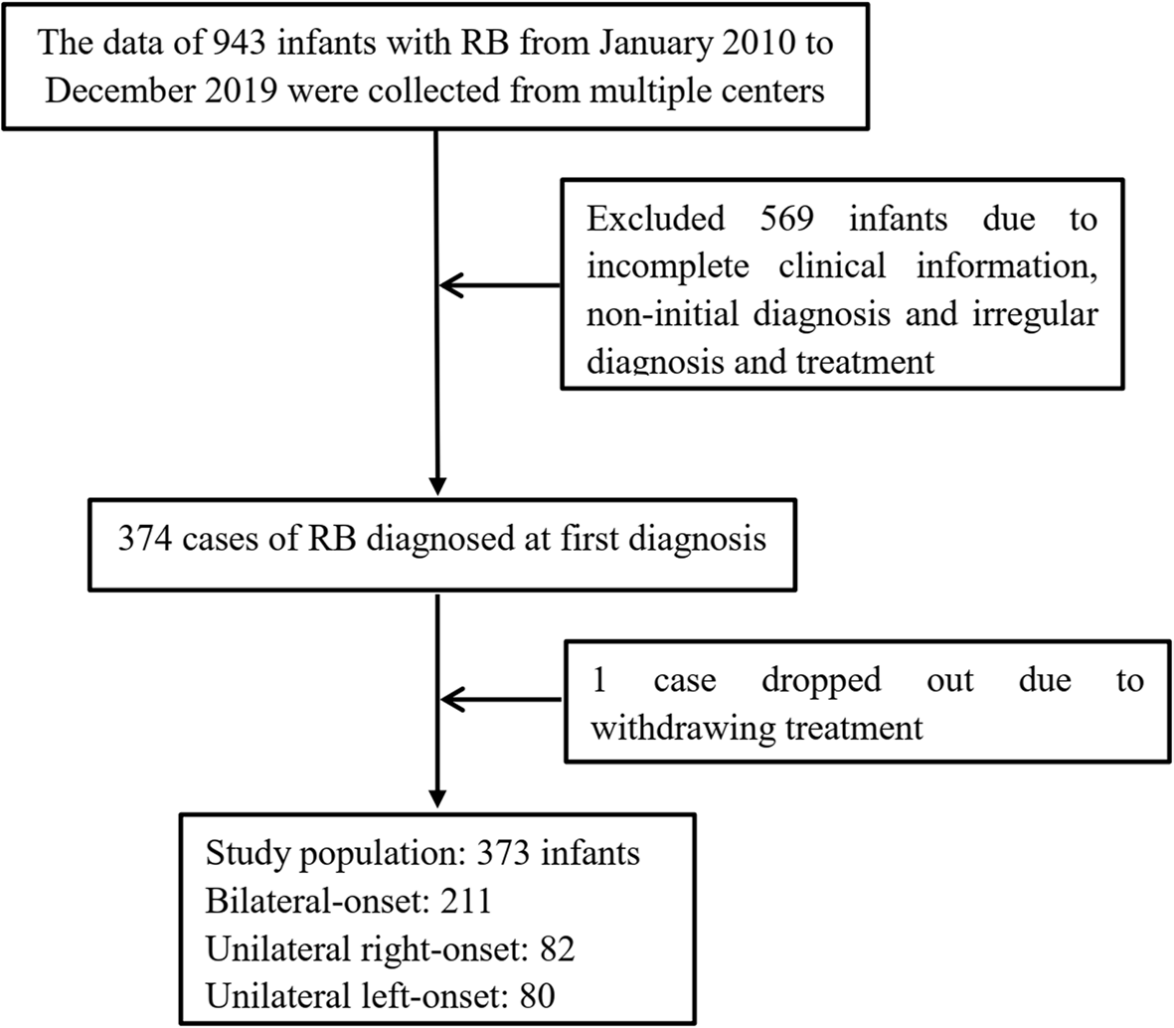
Flowchart of patients’ selection

Among the 943 infants, 569 RB infants were not included in the study due to incomplete clinical information, non-initial treatment and irregular diagnosis and treatment, 1 case dropped out due to withdrawing treatment, finally, 373 infants with RB were included, including 202 boys (54.2%) and 171 girls (45.8%), the median age was 6.22 months (10 days to 11.93 months), of which 180 cases were ≤ 6 months (48.4%) and 193 cases were 7–12 months (51.6%) at diagnosis (Fig. 1). Among the 373 RB infants, a total of 584 eyes were affected (291 left eye and 293 right eye), they consisted of 80 unilateral left-onset of RB, 82 cases (22.0%) unilateral right-onset of RB, and 211 cases (56.6%) bilateral-onset of RB. 17 cases (4.6%) had a family history of RB, 1 case (0.3%) had a family history of other tumors, and 355 cases (94.9%) had no family history. The main presenting sign was leukocoria, accounting for 74.1% (277/373). The most frequent clinical classifications were group D, accounting for 41.6% (121/291) in the left eye and 46.4% (136/293) in the right eye (Table 1).

**Table 1:** Clinical characteristics of RB patients

Characteristics	Value
Gender (n)	373
Boy/Girl	202/171
Age at diagnosis, n (%)	373 (100.0)
< 6 months	180 (48.3)
> 6 months	193 (51.7)
Presenting signs, n (%)	373 (100.0)
Leukocoria	277 (74.1)
Strabismus	26 (7.0)
Infections of the eyeball	23 (6.1)
Physical examination	27 (7.2)
Proptosis	5 (1.3)
Ocular motility disorder	5 (1.3)
Conjunctival hyperemia	7 (1.9)
Other	1 (0.3)
Family history, n (%)	373 (100.0)
RB related	17 (4.6)
Other tumor related	1 (0.3)
No	355 (94.9)
Eye affected	373 (100.0)
Left eye	80 (21.4)
Bilateral eye	211 (56.6)
Right eye	82 (22.0)
Clinical stages	
Left eye	291 (100.0)
Group A	12 (4.1)
Group B	40 (13.7)
Group C	20 (6.9)
Group D	121 (41.6)
Group E	91 (31.3)
Extraocular disease stage	5 (17.2)
Eyeball atrophy or spontaneous regression	1 (0.3)
Unclear	1 (0.3)
Right eye	293 (100.0)
Group A	16 (5.5)
Group B	37 (12.6)
Group C	23 (7.9)
Group D	136 (46.4)
Group E	77 (26.3)
Extraocular disease stage	3 (1.0)
Unclear	1 (0.3)

### Treatment of RB infants

As Table 2 shown, a total of 150 case underwent eye enucleation, including 72 cases of left eye enucleation, 75 cases of right eye enucleation and 3 cases of binocular eye enucleation. The preservation rate of left eye was 74.2% (216/291) and that of right eye was 73.4% (215/293). 44 cases underwent primary enucleation, accounting for 11.8% (44/373), and 106 cases underwent secondary enucleation, accounting for 28.4% (106/373). Among the 8 extraocular cases, 5 cases were performed with enucleation, accounting for 62.5% (5/8), and 145 intraocular cases (39.7% (145/365) were performed with enucleation. Among 373 infants with RB, 368 cases were treated with systematic chemotherapy (98.4%), 57 cases with IAC (15.3%), 67 cases with vitrectomy (18.0%), only 1 case with allogeneic scleral transplantation (0.3%), and 49 cases with intraocular topical therapy (49/373, 13.1%).

**Table 2:** All treatments and prognosis of patients

Group	n (%)	Enucleation rates (%)
Eye enucleation	150 (100.0)	*n* = 373
Left eye	72 (48.0)	52.5 (42/80)
Right eye	75 (50.0)	47.6 (39/82)
Bilateral eye	3 (2.0)	32.7 (69/211)
Systematic chemotherapy	368 (98.7)	40.5 (149/368)
IAC	57 (15.3)	19.3 (11/57)
Vitrectomy	306 (82.0)	45.1 (138/306)
Intraocular topical therapy	49 (100.0)	26.5 (13/49)
Retinal laser photocoagulation	36 (73.5)	22.2 (8/36)
Retinal laser photocoagulation + extrascleral freezing	6 (12.3)	50.0 (3/3)
Extrascleral freezing	5 (10.2)	40.0 (2/5)
Transpupillary thermotherapy	1 (2.0)	0.0 (0/1)
More than two methods	1 (2.0)	0.0 (0/1)
Allogeneic scleral transplantation	1 (0.3)	100.0 (1/1)
Prognosis	374 (100.0)	
Survival	345 (92.2)	41.4 (143/345)
Death	9 (2.4)	22.2 (2/9)
Unclear	20 (5.3)	26.3 (5/19)

*IAC* Intra-arterial chemotherapy

### Prognosis of RB infants

**Fig. 2:**
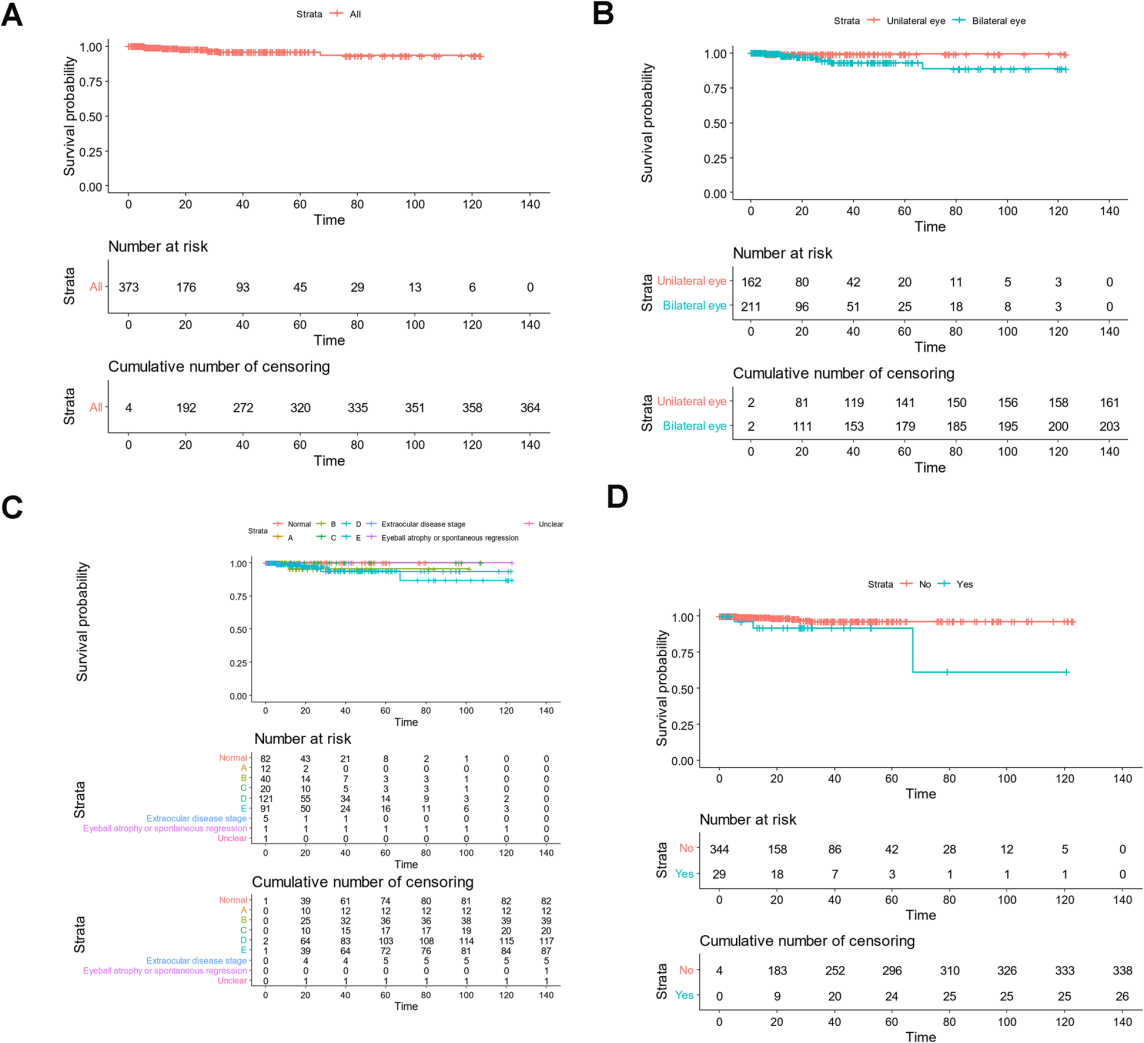
**A** For the 373 RB infants, Kaplan–Meier survival analysis indicated that the median survival time of RB infants was 20.53 months, with 95% CI of 16.969–24.082. **B** Kaplan-merier analysis showed that the OS of infants with binocular disease was lower than that of infants with unilateral eye disease (99.4% vs 96.2%, χ^2^ = 4.188, *P* = 0.041). **C** Kaplan-merier analysis indicated that stage E in left eye had the worst prognosis, which was statistically different from other stages (91.5% *vs* 93.1%, χ^2^ = 5.579, *P* = 0.016). **D** Survival analysis of recurrence and prognosis showed that the prognosis of infants with recurrence was worse than that of infants without recurrence (χ.^2^ = 6.14, *P* = 0.013)

The median follow-up time of RB infants was 18.4 months (1.02–122.81 months). For the 373 RB infants, Kaplan–Meier survival analysis indicated that the 9 cases died and the median survival time was not reached (Fig. 2A). 92.5% (345/373) patients survived, 2.4% (9/373) RB infants died, and 5.3% (20/373) RB infants were lost to follow-up. After excluding the lost to follow-up cases, the OS rate was 97.7% (345/353). The OS rate of infants with unilateral eye affected was significant higher than that of patients with bilateral eye affected (99.4% (161/162) *vs* 96.2% (203/211), *P* = 0.041, Fig. 2B). During follow-up, 29 cases recurred RB with OS rate 79.3% (23/29), 3 cases died, 5 cases gave up treatment, and 23 cases survived. 150 cases (40.2%, 150/373) underwent eye enucleation, including 44 cases (11.8%, 44/373) underwent eye enucleation directly after diagnosis with OS rate 100%, and 106 cases (28.4%, 106/373) underwent eye enucleation after chemotherapy with OS rate 98.1% (104/106), and 223 cases (59.8%, 223/373) did not undergo eye enucleation with OS rate 96.9% (216/223). Statistical analysis showed that eye enucleation and preoperative chemotherapy had no effect on prognosis (*P* = 0.120). Further, the OS of infants with secondary enucleation was 95.3% (101/106), the mean survival time was (32.496 ± 2.883) months, and the 95% CI was 26.846–38.147. There were 143 cases followed up < 12 months. 227, 160, 113, 75 and 46 cases were respectively followed up at 1-, 2-, 3-, 4- and 5-year, and the cumulative OS rate were 98.0%, 96.0%, 96.0%, 96.0% and 92.0%, respectively.

### Analysis of prognostic risk factors

Univariate analysis of prognostic factors revealed eye affected (χ^2^ = 4.19, *P* = 0.041, Fig. 2B), left eye stage (χ^2^ = 5.76, *P* = 0.016, Fig. 2C), recurrence (χ^2^ = 6.14, *P* = 0.013, Fig. 2D) and presenting signs (χ^2^ = 4.18, *P* = 0.041) were poor prognostic factors for OS rate in infants with RB (Table 3). Multivariate Cox regression analyses for OS showed left eye stage (HR = 3.510, 95% CI: 3.409–3.729, *P* = 0.001), recurrence (HR = 1.376, 95% CI: 0.878–2.156, *P* = 0.048) and presenting signs (HR = 2.304, 95% CI: 2.084–2.419, *P* = 0.022) were independent factors for prognosis of infants with RB (Table 3). The median survival time of infants with chemotherapy + IAC + enucleation + vitrectomy was the longest than other groups (*n* = 9, 47.64 months, OS = 100%, all *P* < 0.05). The prognosis of infants with chemotherapy + enucleation + IAC (median survival time, 27.63 months; OS, 50% [1/2]) and infants with IAC alone / + intraocular laser cryotherapy (median survival time, 29.93 months; OS, 95% [19/20]) was relatively lower than that of infants with vitrectomy alone / + intraocular laser cryotherapy (median survival time, 10.42 months; OS, 96.5% [137/142]), chemotherapy + enucleation + vitrectomy (median survival time, 20.53 months; OS, 99.2% [128/129]), chemotherapy + enucleation (median survival time, 22.01 months; OS, 100% [12/12]), IAC + vitrectomy + chemotherapy (median survival time, 22.87 months; OS, 100% [26/26]), or chemotherapy alone / + intraocular topical therapy (median survival time, 28.32 months; OS, 97% [32/33]) (all *P* < 0.05). However, there was no statistical difference in the prognosis of infants among various comprehensive treatment methods (*P* = 0.417).

**Table 3:** Analysis of prognostic risk factors for OS in all patients

Factors	Univariate analysis				Multivariate Cox regression analyses	
	N	OS, n (%)	χ^2^	*P*	HR (95% CI)	*P*
Eye affected	373	364 (97.7)	4.19	0.041	0.206 (0.196–0.257)	0.837
Unilateral eye	162	161 (99.4)				
Bilateral eye	211	203 (96.2)				
Recurrence	29	26 (89.7)	6.14	0.013	1.376 (0.878–2.156)	0.013
Clinical stages of left eye	291	264 (92.7)	5.76	0.016	3.510 (3.409–3.729)	0.001
Group A-C	72	67 (93.1)				
Group D-E	212	194 (91.5)				
Extraocular disease stage	5	3 (60.0)				
Not staged	1	0 (0.0)				
Eyeball atrophy	1	0 (0.0)				
Presenting signs	373	364 (97.7)	4.18	0.041	2.304 (2.084–2.419)	0.022
Leukocoria	277	259 (93.5)				
Vision loss and blindness	6	6 (100.0)				
Proptosis	2	2 (100.0)				
Strabismus	26	24 (92.3)				
Ocular motility disorder	5	5 (100.0)				
Conjunctival hyperemia	7	7 (100.0)				
Infections of the eyeball	23	20 (90.0)				
Physical examination	27	22 (81.5)				

*IAC* Intra-arterial chemotherapy

### Prognostic analysis of infant with familial RB

**Fig. 3:**
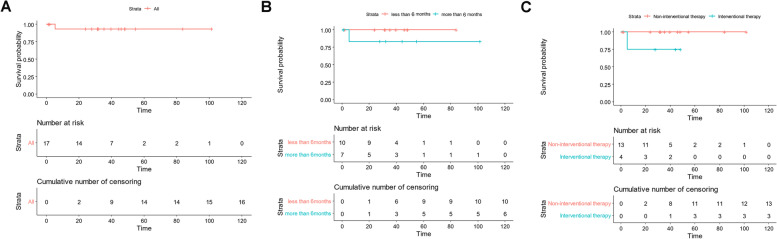
**A** Kaplan-merier survival analysis curve of 17 familial infant RB, the median survival time was 39.5 months (95% CI 25.117–53.8). **B** Among the 17 cases of familial infant RB, the prognosis of children younger than 6 months of age was similar to that of the infant 6–12 months of age (*P* = 0.22). **C** The OS of 17 familial infants RB with interventional therapy was lower than that of infants with non-interventional therapy (*P* = 0.049)

There was a history of RB in 17 infants' lineal relatives, including 8 boys (47.1%) and 9 girls (52.9%). 10 cases (58.8%) were aged < 6 months, 7 cases (41.2%) were aged from 6 to 12 months. There were 14 (82.4%) cases of binocular disease, 3 (17.6%) cases of unilateral eye disease, and 31 eyes of IIRC stage, including 14 eyes of group D + E (45.2%), 1 eye of extra-ocular stage, 16 eyes of group A-C (51.6%). 6 infants underwent monocular excision, 3 infants underwent vitrectomy, 6 infants received chemotherapy, and 4 infants received interventional therapy. After follow-up, 16 cases with event free survival and 1 case died (recurrence after intervention followed by progression of orbital and intracranial disseminated tumors). During the follow-up, 3 cases recurred, 1 case died of tumor progression, and 2 cases achieved event free survival after chemotherapy and surgery. Kaplan-merier survival analysis indicated that 1 case died and the median survival time was not reached (Fig. 3A). Univariate analysis of survival showed that the prognosis of infants < 6 months was similar to that of infants aged 6–12 months (*P* = 0.22, Fig. 3B), and the prognosis of infants without interventional therapy was better than that of infants with interventional therapy (*P* = 0.049, Fig. 3C). Multivariate analysis showed interventional therapy was not an independent risk factor.

## Discussion

RB is the most common intraocular malignancy in childhood, can present in one or both eyes, which seriously endangers children’s vision and life [17]. The clinical symptoms of RB patients were leukocoria, strabismus, lachrymation, iris turn colors, pinkeye, forward antrum bleeding, orbital inflammation, glaucoma and nubecula, among which leukocoria is the most common initial presenting sign in infants [7]. It is worth mentioning that leukocoria is not only seen in RB patients, but also in pseudoretinoblastoma, including Coats' disease, persistent fetal vasculature (PFV), vitreous hemorrhage, familial exudative vitreoretinopathy (FEVR) and other eye diseases [18–20]. Therefore, we recommend that when examining infants with possible RB consult RB specialist before planning treatment strategies. Our study may be the first and largest clinical date of RB infants (≤ 12 months) in China. In this study, 74.1% of the infants had the leukocoria as initial presenting sign, which was consistent with the literature report, followed by strabismus, decreased vision, conjunctival hyperemia and infection. A small number of children with unilateral RB later develop metachronous bilateral RB [21]. One previous study reported that the average age at the time of RB diagnosis is 23 months, the age of onset of RB in bilateral eyes was 15 months, which was smaller than the age of onset of RB in unilateral eyes at 27 months [9]. In our study, the incidence of RB in bilateral eye was higher than that in unilateral eye (56.6% vs 43.4%), and the incidence of RB in infants aged 6–12 months was slightly higher than that in children aged < 6 months (51.6% vs 48.4).

The cure of RB and the sequelae arising from RB mainly depend on an early diagnosis [22]. Tracing family history and paying attention to birth history during pregnancy are very helpful for early diagnosis of RB. By analyzing the morbidity characteristics of medical records, it suggests that not only the family history of RB should be included in the neonatal ophthalmic screening, but also the history of immediate relatives (congenital eye disease or unexplained weakness, myopia and blindness) should be included in the screening [23]. Meanwhile, it is suggested infant with low birth weight or abnormal birth should be conducted in early screening. In our study, it was worth noting that 7.2% (27/373) cases were clinically diagnosed with RB through physical examination and screening, and even higher than the proportion of strabismus (7.0%, 26/373). These findings indicated that the early screening for patients with family high-risk factors or high-risk factors in birth history was conducive to the early discovery and diagnosis of RB.

Clinical studies show that for early stage patients, conservative treatment can be optional with follow-up of retinal tumor morphologic changes [24]. One study has indicated that, the treatment method could be selected according to the degree of progress of the tumor for D stage patients, in order to maximize patients’ life quality. The proposal to E stage patients was enucleation to prevent recurrence and metastasis [25]. In our study, 584 eyes were affected. According to the IIRC stage, the stages of tumor space occupying in the left and right eyes were mainly D and E stages, which were 71.9% (41.6% + 31.3%) and 68.7% (46.4% + 26.3%) respectively. Survival analysis showed that the prognosis of stage E infants was worse than that of other stages. There was no consistent conclusion at home and abroad whether it indicated that the condition of RB infant was serious. In this study, infants with intraocular phase E, especially those within 6 months, often chose to delay enucleation after chemotherapy and volume reduction because they were unable to install artificial eyes immediately after enucleation. Statistical analysis that preoperative chemotherapy followed by eyeball enucleation did not affect the prognosis of stage E and extraocular children. Furthermore, it is worth noting that the prognosis of children with vitrectomy is worse than that of children without vitrectomy, so the indications should be more cautious.

The diagnosis level, average age of onset and prognosis of RB patients vary slightly in different countries and regions. The mortality rate of RB in most developed countries such as Europe is lower than that in developing countries [26]. The treatment principle of RB is to preserve life first, and then to use a variety of diagnosis and treatment methods to preserve eye organs and functions as much as possible on the premise of preserving life [2]. In the United States, 75% of children with advanced or refractory RB, who cannot be treated with focal therapy alone, may undergo systemic chemotherapy, external beam radiotherapy, or enucleation [27]. In this study, intrathecal therapy was added for RB infants with definite retrobulbar optic nerve invasion and extraocular phase. Moreover, due to the young age of onset and the high dispersion of local drugs, IAC also brought a high risk of local side effects, thus systemic chemotherapy was as the main treatment method, instead of IAC. A number of studies at home and abroad have reported that intra-arterial chemotherapy and/or intravitreal chemotherapy are the main diagnosis and treatment scheme for RB, and stem cell transplantation index is also recommended for distant tissue metastasis. According to the those reports, children under 2 years of age have also achieved a good rate of eyeball preservation, with the enucleation rate of about 43%, and the recurrence rate of less than 25% [15, 28, 29]. In this study, the rate of eyeball extraction was basically the same as reported or slightly lower than that reported in the literature, and the recurrence rate was only 7.8% (29/373). During follow-up, 9 cases died, 20 cases fell out, 345 case survived, and the OS was high as 97.7% (345/353), which was similar to the report of Tamaki et al. (OS rate, 94.9%) [8]. The OS rate of patients with unilateral or bilateral eye affected was 99.4% (161/162) and 96.2% (203/211), respectively, indicating that the prognosis of OS in infants with unilateral RB was higher than that with bilateral RB.

It is clear that RB is a prototypical genetic cancer, which is related to the mutation of RB1 gene located in the long arm of chromosome 13 (13q14) [30]. The occurrence of RB has a clear tendency, mainly including germline inheritance and non-germline inheritance. RB is heritable in 40% of the cases and, in such cases, tumors are bilateral in 80%, unilateral in 15%, and trilateral in 5% of the cases [31]. In this study, familial RB germline genetic pattern accounts for 4.6% (17/373), and the occurrence of RB in other children may be related to secondary mutation [32]. Moreover, 82.4% (14/17) of the 17 cases of familial RB infant have binocular disease, which was consistent with previous report. Rare deaths in our study might be related to the probable germline defect of RB1 gene, although most patients received no genetic study. To pursue a better quality of life, neonatal screening is being more actively promoted for high-risk families in China under collaboration between ophthalmologists and pediatricians. Moreover, in familial RB, the only death case was intracranial spread death caused by recurrence and progression after interventional therapy, indicating that intraocular surgery and interventional chemotherapy should pay attention to the prevention and treatment of the indication and postoperative prognosis of tumor spread caused by arteriovenous.

## Conclusions

In general, the onset of RB in infants was early, and the onset can occur as early as a few days after birth. Recurrence is an independent factor for prognosis of RB infants, which still needs attention after treatment. Although some of the infants had a clear family history, the prognosis was high after comprehensive diagnosis and treatment, reaching 97.7%. Therefore, early diagnosis/screening and comprehensive treatments should be adhered to in order to achieve long-term survival of RB infants.

## Data Availability

Due to institutional ethics restrictions, the dataset of the patients supporting the current study has not been deposited in a public repository, but is available from the corresponding authors upon request.
